# Local delivery of decorin via hyaluronic acid microrods improves cardiac performance and ventricular remodeling after myocardial infarction

**DOI:** 10.21203/rs.3.rs-2501087/v1

**Published:** 2023-02-08

**Authors:** Tejal Desai, Priya Mohindra, Justin Zhong, Qizhi Fang, Cindy Huynh, Darnell Cuylear, Huiliang Qiu, Dongwei Gao, Bhushan Kharbikar, Xiao Huang, Matt Springer, Randall Lee

**Affiliations:** University of California, San Francisco & Brown University; University of California, Berkeley & University of California, San Francisco; University of California, Berkeley & University of California, San Francisco; University of California, San Francisco; Brigham and Women’s Hospital, Harvard Medical School; University of California, San Francisco; University of California, San Francisco; University of California, San Francisco; University of California, San Francisco; University of California, San Francisco; UCSF; University of California, San Francisco

## Abstract

Heart failure (HF) is a global public health burden and associated with significant morbidity and mortality. HF can result as a complication following myocardial infarction (MI), with cardiac fibrosis forming in the myocardium as a response to injury. The dense, avascular scar tissue that develops in the myocardium after injury following MI creates an inhospitable microenvironment that hinders cellular function, survival, and recruitment, thus severely limiting tissue regeneration. We have previously demonstrated the ability of hyaluronic acid (HA) polymer microrods to modulate fibroblast phenotype using discrete biophysical cues and to improve cardiac outcomes after implantation in rodent models of ischemia-reperfusion MI injury. Here, we developed a dual-pronged biochemical and biophysical therapeutic strategy leveraging bioactive microrods to more robustly attenuate cardiac fibrosis after acute myocardial injury. Incorporation of the anti-fibrotic proteoglycan decorin within microrods led to sustained release of decorin over one month in vitro and after implantation, resulted in marked improvement in cardiac function and ventricular remodeling, along with decreased fibrosis and cardiomyocyte hypertrophy. Together, this body of work aims to contribute important knowledge to help develop rationally designed engineered biomaterials that may be used to successfully treat cardiovascular diseases.

## Introduction

Heart failure (HF) affects approximately 6 million Americans, and the prevalence is projected to increase 46% from 2012 to 2030.^1^ The prognosis for HF is poor, with an estimated mortality rate of ≈ 50% within 5 years of the diagnosis.^1^ Myocardial infarction (MI) from coronary artery disease is the leading cause of HF and despite advances in the management of MI, subsequent pathologic remodeling of ischemic myocardium with fibrotic scar tissue and aneurysmal degeneration leads to HF and death.^2^ Therefore, therapies that can prevent scar tissue formation, increase cell survival, and promote contractile tissue regeneration need to be identified to treat this growing patient population in the wake of increasing rates of obesity and heart disease.^1^ To date, cellular therapies have had limited success in promoting long-term cardiovascular repair^3,4^ and no delivery system for an effective pharmacotherapy exists. Moreover, current treatment of fibrosis involves systemic inhibition of cytokines and chemokines which leads to many adverse side effects for patients.^5,6^

In recent years, there has been growing interest in targeting pathways that lead to altered cardiovascular cell phenotypes and microenvironments after injury to reduce maladaptive repair and promote functional recovery. As most of these cell types are mechanosensitive and rely on micro- and nanoscale cues from the extracellular matrix (ECM) to dictate homeostatic function, it is possible to harness these interactions through biomaterials with similar size scale biophysical cues to elicit more native cell phenotypes, thereby mitigating cardiovascular disease progression and enhancing regenerative potential.^7^ Fibroblasts represent the largest percentage of cells in the heart and coordinate numerous functions including ECM turnover, cell-cell signaling, and cytokine and growth factor secretion.^8^ After MI, fibroblasts transform into a highly contractile, activated myofibroblast phenotype, which functions to stabilize the injury site by increasing ECM deposition to preserve the integrity of the myocardial wall and maintain the pressure generating ability of the heart.^9^ While this compensatory process is initially beneficial, problems arise when pro-fibrotic inflammatory signals such as TGF-β1 persist, leading to continual deposition of stiff scar tissue which ultimately impairs contractility and perfusion within in the heart.^9^

We have previously demonstrated the ability to modulate fibroblast morphology and function using polymeric microstructural cues to achieve less fibrotic phenotypes, which could have tremendous implications in heart failure therapy.^10–13^ Recent work showed that in vitro treatment with polymeric microrods (15 × 15 × 100 μm) decreased fibroblast proliferation and that microrod injections in preclinical rodent models of heart failure cause reductions in scar tissue and improvements in cardiac function by influencing the cardiac microenvironment.^12,13^ Benefits of using hydrogel microstructure strategies with mechanobiological mechanisms of action as therapeutic approaches include being injectable, cell-free, and highly tunable in terms of geometry, stiffness, and material. Further, as their therapeutic effects are restricted based on proximity, concerns related to systemic side effects are avoided. Microrod hydrogels also allows for combination therapies with the ability to load and release various therapeutic factors from the microstructures.^14,15^ The ability to devise more potent, multi-faceted therapies can have tremendous implications on myocardial regeneration after MI by addressing multiple pathological processes.

Reperfusion strategies are a key component in the management of acute MI but can lead to ischemia reperfusion injury (IRI), which is widely characterized by oxidative stress, inflammation, intracellular Ca^2+^ overload, fibrosis, and endothelial dysfunction.^16–18^ Although the exact mechanisms of IRI remain unknown, targeting oxidative damage and subsequent fibrotic response that occurs after myocardial injury and reperfusion is critical to cardiac recovery after MI.^19–26^ Several naturally occurring biological macromolecules within the body possess unique characteristics that may enhance intrinsic wound healing functions after injury. Small leucine rich proteoglycans (SLRPs) are ubiquitous ECM components involved in structural organization and are known regulators of collagen fibril assembly.^27^ Decorin, a class I SLRP has been shown to have both antifibrotic and antioxidant properties. It has been reported to sequester the profibrotic cytokine TGF-β with high affinity and modulate collagen fibrillogenesis.^28–34^ Preclinical studies have also demonstrated a therapeutic role of decorin in mitigating fibrosis in various in vivo models of ischemic injury^35–37^ and in vitro fibrosis models.^38–40^ Further, it has been reported that there is a protective role of decorin after traumatic injury in vivo which is linked to oxidative stress response.^35,41^ In cell studies with high glucose and oxygen/glucose-deprived environments, protective effects of decorin center on involvement in apoptosis and oxidative stress pathways.^42,43^ Therefore, there may exist an important role for decorin in early response therapies for cardiovascular injury.

Hence, our approach in this study is to introduce bioactive, decorin-loaded hyaluronic-acid (HA) microstructural cues into the post-infarct microenvironment to regulate pathological cell responses and transform the wound healing microenvironment by providing discrete micromechanical and biochemical cues. We demonstrate the impact of decorin-loaded microrods on cardiac function, ventricular remodeling, fibrosis, hypertrophy, and vascularization post-MI.

## Materials And Methods

### 1.3.1. Materials

Sodium hyaluronate (100kDa) was obtained from Lifecore Biomedical and stored at −20°C until use. Dimethylformamide (DMF), glycidyl methacrylate (GM), triethylamine (TEA), 2-hydroxy-4’-(2-hydroxyethoxy)-2-methylpropiophenone (#410896), differentiation solution (A3179-1L), 2-methylbutane, picric acid solution (P6744), Fast Green FCF (F7252), and Direct Red 80 (#365548) were purchased from Sigma-Aldrich (St. Louis, MO) and used as received. Spectrum^™^ Spectra/Por^™^ 3 RC dialysis membrane tubing, 3500 Dalton MWCO (#08-670-5B), Wheat Germ Agglutinin (WGA) Alexa Fluor 488 (#W11261), Hoescht (#33342), high concentration rat tail collagen type I (#CB354249), and NanoOrange Protein Quantitation Kit (#N6666) were purchased from Thermo Fisher Scientific (Waltham, MA). Protein LoBind microcentrifuge tubes (#022431102) were purchased from Eppendorf (Hamburg, Germany). OCT was obtained from Sakura Finetech USA, Inc. (Torrance, CA). Harris Hematoxylin Stain (#95057-858) and Eosin Y Solution (#95057-848) were purchased from VWR (Radnor, PA). Recombinant human TGF-β1 (#100 - 21) was obtained from PeproTech. Recombinant human decorin protein (ab167743), human decorin ELISA kit (ab99998), anti-decorin antibody (ab151988), anti-sarcomeric alpha actinin (ab137346), and anti-alpha smooth muscle actin (ab5694) were purchased from Abcam (Cambridge, UK).

### 1.3.2. Synthesis of hyaluronic acid methacrylate

Hyaluronic acid methacrylate (HAMA) was synthesized based on a protocol adapted from Bencherif *et al*.^44^ These methods have been previously reported by our lab.^13^ Briefly, one gram of sodium hyaluronate (100 kDa) was dissolved at 3.76 mg/mL in a 1:1 solution of deionized (DI) water:DMF (266 mL). After solubilizing, a 73-fold molar excess of GM (24.86 mL, 0.1822 mol) and 26.5-fold molar excess of TEA (9.235 mL, 0.066 mol) with respect to the primary hydroxyl/hydroxymethyl functional group on hyaluronic acid was added to the mixture. The reaction was left to stir for 24 hours at ambient temperature while protected from light. HA and HAMA products were recovered via precipitation in an excess of isopropanol. Briefly, 35 mL of isopropanol was added to 15 mL of reaction solution and then the precipitate was isolated by centrifugation at 1275 × g for 5 min. This process was repeated until all of the reaction solution had been precipitated. The recovered precipitate was subsequently dissolved in 90 mL of DI water. The resulting solution was then dialyzed (3500 Dalton MWCO) against DI water (10 times volume of the solution) for 48 hours with three changes of water. The product was then lyophilized for 3 – 4 days at − 40°C and 65 mTorr and the resulting white powder was then stored at − 20°C until further use. ^1^H NMR spectroscopy (Bruker Avance III HD 400 NMR) was used to determine the degree of methacrylation. Methacrylate peaks are observed at ~ 6.5, ~ 5.6, and ~ 1.85 ppm. Degree of methacrylation was calculated based on the ratio of the relative peak integration of the methacrylate peak at 1.85 ppm and HA’s acetamide peak which occurs at 1.9 ppm and was determined to be approximately 50.1 ± 6.9% substitution (data not shown).

### 1.3.3. Hyaluronic acid microrod fabrication

**Figure 1:**
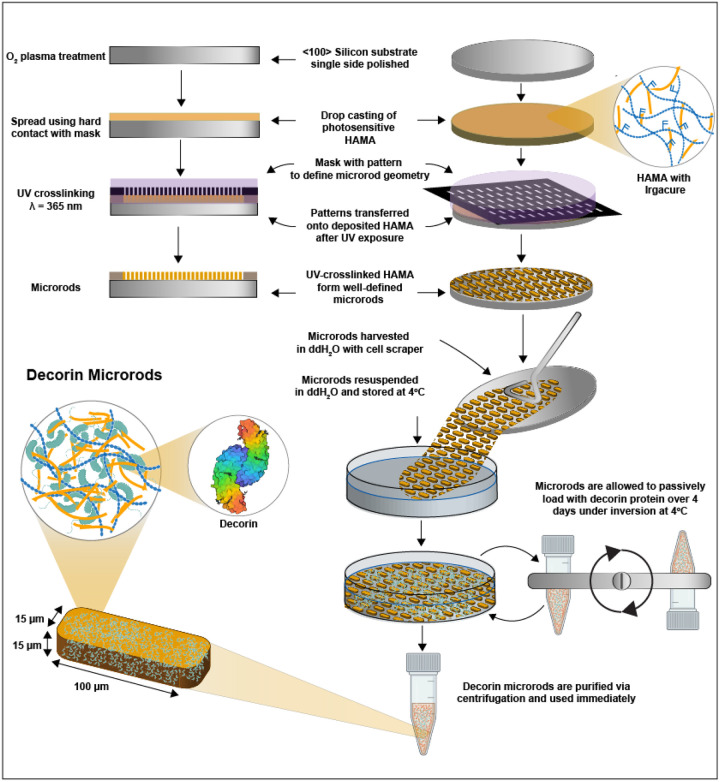
Microrod fabrication scheme and loading with decorin. HA was first modified with a photosensitive handle to generate hyaluronic acid methacrylate (HAMA) polymer that is amenable to crosslinking via exposure to UV light in the presence of the photoinitiator 2-hydroxy-4’-(2-hydroxyethoxy)-2-methylpropiophenone. HAMA was deposited onto an oxygen plasma- or piranha-treated wafer and exposed to UV light through a photomask patterned with fixed rectangular geometries of 15 μm width by 100 μm length. After exposure, the polymer film was then developed in water to isolate the crosslinked microrods. Microrods were loaded with decorin via incubation with inversion at 4°C over 4 days and then subsequently purified via centrifugation.

HAMA was dissolved at 75 mg/mL in DI water containing 0.5% w/v of the photoinitiator 2-hydroxy-4’-(2-hydroxyethoxy)-2-methylpropiophenone. The solution was stirred for 2 hours at ambient temperature to facilitate solubilization and protected from light. After, the solution was centrifuged at 15000 × g for 5 minutes to remove any impurities. Subsequently, a 15 μm layer of precursor solution was deposited onto an oxygen plasma- or piranha-treated 3” silicon wafer. The wafer was then patterned using a Karl Suss MJB3 or Quintel Q4000 mask aligner by exposing the wafer through a photomask (15 μm × 100 μm features) to a 365 nm UV light source. Crosslinked microrods were then gently removed from the wafer using a cell scraper and collected into DI water, where any uncrosslinked HAMA would fully dissolve. Microrods were then passed through a 150 μm mesh filter to remove any aggregates and then concentrated by centrifugation. The microrods were subsequently sterilized with 70% ethanol for 30 minutes and then resuspended in saline prior to use. Microrod concentration was determined using a hemocytometer by counting the number of microrods that appeared in the nine gridded boxes after pipetting 10 μL of microrod solution into the hemocytometer. The number of counted microrods was then divided by 9, and then multiplied by 10^4^ and the dilution factor to get the microrod concentration in the stock solution. The microfabrication process is illustrated in Fig. 1.

### 1.3.4. Decorin loading of microrods

To passively load microrods with decorin, 750,000 microrods were concentrated in 1 mL of deionized (DI) water and then 333 μL of decorin (600 μg/mL) was added. The microrods were passively loaded via incubation with inversion over four days at 4°C. After incubation, the microrods were centrifuged at 15.000 × g for 10 minutes and the supernatant was removed. The microrods were then recentrifuged at 15.000 × g for 10 minutes once more to concentrate them and remove any residual solution. The microrods were then resuspended in saline to the desired concentration. Decorin loading was assessed via NanoOrange Protein Quantitation Kit (Thermo Fisher, Waltham, MA). Values were validated with an ELISA for human decorin (Abcam, Cambridge, UK) (data not shown). Decorin loading was determined by calculating the amount of free decorin in the supernatant post-centrifugation, and then subtracting that value from the initial amount of decorin added (200 μg), and then dividing that value by 750,000 to get the amount of decorin μg) per microrod.

### 1.3.5. Decorin release study from microrods

After loading, decorin microrods were divided into Protein Lo-Bind tubes (Eppendorf, Hamburg, Germany) consisting of 50,000 microrods/microcentrifuge tube, and then suspended in 1 mL of phosphate buffered saline (PBS) containing 0.05% Tween-20 (pH 7.4). Samples were placed onto a tube revolver (14 RPM) within an incubator (37°C). Decorin microrods were spun down at 8,000 RPM for 3 minutes and the entire supernatant was collected and replenished with 1 mL at 4, 8, 16, 24, 72, 96, 120, 144, 168, 240, 336, 432, 504, and 744 hours. Collected supernatants were immediately flash frozen and stored at − 80°C until further use. An ELISA for human decorin (Abcam, Cambridge, UK) was performed per the manufacturer’s instructions and hourly release amounts were calculated using an established standard curve using human decorin (Abcam, Cambridge, UK).

### 1.3.6. Collagen turbidity assay

First, a 1 mg/mL stock solution of collagen type I was prepared by dilution of high concentration collagen type I with PBS and was subsequently kept on ice along with a 96-well plate. Varying decorin concentrations ranging from 5–80 μg/mL were then prepared by diluting 600 μg/mL stock decorin solution with PBS. Then, a 1:1 ratio of 1 mg/mL collagen type I and the decorin stocks were added to each well in triplicate resulting in final decorin doses of 2.5, 5, 10, 20, and 40 μg/mL. Absorbance measurements at 405 nm were then taken every 5 min over the span of 2 hours at ambient temperature using a plate reader. The assay also included blank controls of water and PBS as well as a control for the highest concentration of decorin (40 μg/mL).

### 1.3.7. Cell culture & qCPR

NIH 3T3 mouse fibroblasts (ATCC, Manassas, Virginia) were cultured in Dulbecco’s modified Eagle’s medium with 10% fetal calf serum and 1% penicillin/streptomycin. Cells were seeded into 24-well plates and then media was added that either contained only 10 ng/mL TGF-β1 or 10 ng/mL TGF-β1 plus 10 μg/mL decorin. Genetic material was harvested and purified using the RNeasy Mini Kit (Qiagen, Hilden, Germany). RNA was converted into cDNA using the iScript cDNA synthesis kit (Bio-Rad Laboratories, Hercules, CA) and a Viia7 qPCR machine (Life Technologies, Carlsbad, CA) was used to measure relative expression levels of gene targets compared to the housekeeping gene 60S ribosomal protein L19 (RPL19). Expression levels of all genes were evaluated using the Fast SYBR Green Mastermix (Life Technologies, Grand Island, NY) and custom DNA primers (Integrated DNA Technologies, Coralville, IA) in triplicate for three biological replicates (Table S1).

### 1.3.8. Infarct model and microrod injections

The animal protocol for MI induction was approved by the Committee for Animal Research of the University of California, San Francisco and was performed in accordance with the recommendations of the American Association for Accreditation of Laboratory Animal Care. The ischemia reperfusion (I/R) MI model used in this study has been extensively tested and successfully used in our labs.^12,13,45,46^ To produce the MI model, male Sprague-Dawley rats (200–225g) underwent occlusion of the left anterior descending coronary artery for 30 minutes followed by reperfusion while under general anesthesia which was achieved by inhalation of 2% L/min isoflurane.^45^ The chest was then sutured closed, and the animal was allowed to recover. After 3–4 days, the rats were randomized to saline-injected, microrod-injected, decorin microrod-injected, or free decorin-injected treatment groups (Table 1), and were given one intramuscular injection into the myocardial wall via ultrasound guided transthoracic injection using a 29-gauge syringe under blinded conditions.^12,13,45,47^ Each injection consisted of 50 μL of sterile 0.9% sodium chloride solution (n = 5), 50 μL of 12 μg of decorin in 0.9% sodium chloride solution (n = 4), 50 μL of 50,000 microrods in 0.9% sodium chloride solution (n = 7), or 50 μL of 50,000 decorin microrods in 0.9% sodium chloride solution (n = 10). Successful injection to the center of the infarct region was confirmed by a local increase in ultrasound signal and brief thickening of the left ventricle (LV) wall near the tip of the syringe.

### 1.3.9. Echocardiography

Transthoracic echocardiography was performed with a 15-MHz linear array transducer system (Vevo 3100LT, FUJIFILM VisualSonics, Ontario, Canada) on all animals while under general anesthesia (2% L/min isoflurane). Echocardiography was performed prior to injection on day 3 or 4 post-MI and eight weeks post-injection using standard methods that have been performed reproducibly in our lab.^12,13,45,46^ To determine left ventricular end diastolic volume (LVEDV), left ventricular end systolic volume (LVESV), and ejection fraction (EF) at 3–4 days and eight weeks, the left ventricular endocardium was outlined in both the end-systolic and end-diastolic phase and the single plane area length algorithmic method was applied. Two-dimensional images were obtained in both parasternal long- and short-axis views at the papillary muscle level. Stroke volume (SV) was calculated by SV = LVEDV-LVESV, while change in EF was calculated by ΔEF = EF_8 weeks_-EF_3–4 days_. All image and subsequent analyses were performed in a blinded manner. In situations where the ventricular endocardium was not clearly identifiable in the 3–4 day or eight-week image, the animal was excluded from echocardiographic analyses. Cases where ejection fraction was above 45% at 3–4 days were excluded because they indicated an insufficient infarct model.

### 1.3.10. Histology

Sacrifice was performed after eight weeks after performing endpoint echocardiography. The animal was maintained at 5% L/min isoflurane for five minutes, followed by bilateral thoracotomy and injection of potassium chloride into the right atrium to arrest the heart in diastole. The heart was then extracted and frozen in OCT (Sakura Finetech USA, Inc., Torrance, CA) on 2-methylbutane (Sigma Aldrich, St. Louis, MO) on dry ice and sectioned for histology, immunofluorescent imaging, and image analysis. Tissue blocks were cryo-sectioned at a thickness of 10 μm starting at the apex of the LV, collecting 10 serial sections every 350 μm until a total of 100 sections were collected. Sections were stained with H&E and Sirius red using standard protocols. Briefly for Sirius red staining, frozen sections were brought to room temperature, soaked in xylene for a total of 15 min., rehydrated using an ethanol series (100%, 95%, and 80%) for 1 min. each, and then soaked in water. Sections were then incubated in 0.01 % Fast Green FCF solution in saturated picric aqueous solution for 1 hr. followed by a 1 hr. incubation in 0.04% Fast Green FCF/0.1% Sirius red in saturated picric acid solution. Next, sections were dipped in acidified water, 100% alcohol, and then xylene prior to mounting. For H&E staining, slides were soaked in tap water for 5 min. to remove OCT from the sections. They were then stained in Harris’s Hematoxylin for 5 min., followed by dipping in water for 1 min., and then dipping in Differentiating Solution for 1 second for a total of 3 times. Next, slides were immersed in Bluing Solution (0.1 % sodium bicarbonate, pH ~ 8.0) for 30 seconds, followed by dipping in water for 1 min. Slides were then soaked in 70% ethanol for 1 min. and then in Eosin Y for 45 sec. Sections were then dehydrated using an ethanol series (85%, 95%, 100%) for 1 min. each before soaking in xylene and mounting. For immunofluorescent stains, tissue sections were air dried and blocked with 10% serum, followed by incubation with primary and secondary antibodies using standard protocols. In brief, samples were fixed in ice cold acetone for 5 min., blocked with 10% serum for 20 min., and incubated overnight with primary antibodies at 4°C in a solution of 0.05% Tween-20, 10% serum, and 1 % BSA in PBS (anti-sarcomeric alpha actin 1:500, anti-alpha smooth muscle actin 1:500). After washing, secondary antibody and WGA (1:100) was added for 45 minutes at room temperature. Hoechst (1:500) was added for 5 min at room temperature to visualize nuclei.

### 1.3.11 Imaging and quantification

For whole heart histology, images were taken using a Nikon 6D optical microscope (NIKON Instruments, Inc., Melville, NY) using 4–40x objectives (H&E and Sirius red). Subsequent quantifications were performed using custom scripts and ImageJ.

For collagen analysis, five alternating sections of each heart were selected from throughout the coronal plane of the heart and stained with Sirius red to assess the quantity and density of collagen in the injured hearts. These sections were imaged under brightfield as well as under cross-polarized light to visualize the collagen fibers using a 4x objective. Representative images for brightfield and polarized shading corrections were taken prior to the start of each imaging session. Infarct area and intensity was quantified in the LV, which included the septum. Intensity measurements in the regions of interest were determined using ImageJ.

For wall thickness analysis, five measurements across the LV free wall were recorded for each tissue section where the LV free wall was distinguishable. The average length across all analyzed sections in each heart sample was reported.

For cardiomyocyte cross-sectional area analysis, three sections (apex, middle, and end of the heart) per heart (n = 4 per group) were selected and stained with anti-sarcomeric alpha actinin, WGA, and Hoechst. Four high-magnification (40x objective) images of the border zone and remote regions in each section, for each animal were averaged to obtain the cross-sectional area, cells per area, eccentricity, major axis length, and minor axis length. Specifically, WGA-stained sections aided in the determination of number of cells per area and were quantified in Python (3.9.7) using OpenCV (4.5.5.64) and scikit-image (0.18.3) packages to conduct watershed thresholding of cell bodies and subsequent area measurements.^48,49^ Images were inverted and then binarized using an adaptive mean threshold before applying the watershed segmentation algorithm. The area of segmented cells, cell count per area, eccentricity, major axis length, and minor axis length were then computed. Segmented images were then manually checked to ensure good quality segmentation, with those showing a high error rate excluded (e.g., high fractionation of cells into multiple segments, doublet or multiplet cells recognized as one cell body, membrane stains segmented as cells). For each image, the average cell area, number of cells per μm^2^, eccentricity, major axis, and minor axis are reported.

For vascular density analysis, three sections (apex, middle, and end of the heart) per heart (n = 4 per group) were selected and stained with anti-alpha smooth muscle actin and Hoechst. Four high-magnification (40x objective) images of the remote, infarct, and border zone regions in each section, for each animal were averaged to obtain the number of arterioles per field. Only vessels that stained positively for alpha smooth muscle actin and possessed a visible lumen were included in the analysis.

### 1.3.12 Statistical analysis

All data are presented as the mean ± standard deviation unless otherwise indicated. In vitro analysis was performed using a student’s t-tests to identify statistical differences between two groups. One-way analysis of variance (ANOVA), followed by the Tukey’s multiple comparisons test was used to identify differences between three or more groups, unless otherwise stated. Statistical comparisons made between groups to assess differences in EF, LV end systolic volume (ESV), and LV end diastolic volume (EDV) at eight weeks post-MI were made using a Two-way repeated measures ANOVA followed by the Tukey’s multiple comparisons test.

## Results

### 1.4.1. Decorin treatment reduces myofibroblast-like gene expression and inhibits collagen fibrillogenesis

To confirm anti-fibrotic biologic effects of decorin, NIH 3T3 fibroblasts were stimulated with 10 ng/mL TGF-β1 and cultured in the presence or absence of 10 μg/mL decorin. Relative gene expression results from RT-qPCR using primers specified in Table 1 indicate stark reductions in ACTA2 (*p*< 0.0001), COL1A2 (*p*< 0.01), COL3A1 (*p*< 0.001), and MMP2 (*p*< 0.05) expression in decorin-treated samples compared to control (Figure S1A-C). These results demonstrate decorin’s ability to sequester TGF-β1 and prevent activation of its downstream signaling pathways that result in myofibroblast-like phenotypes in fibroblasts.

Turbidity was used to assess the degree of collagen fibrillogenesis. A broad range of decorin concentrations (2.5–40 μg/mL) were used to assess dose-dependent effects of decorin on collagen fiber formation. It is evident that fibril formation kinetics are dependent on decorin concentration, and while all doses of decorin tested significantly inhibited fibrillogenesis, the highest concentration of 40 μg/mL yielded the greatest impact (Figure S2, *p*< 0.01).

### 1.4.2. Sustained release of decorin is achieved from microrods

**Figure 2:**
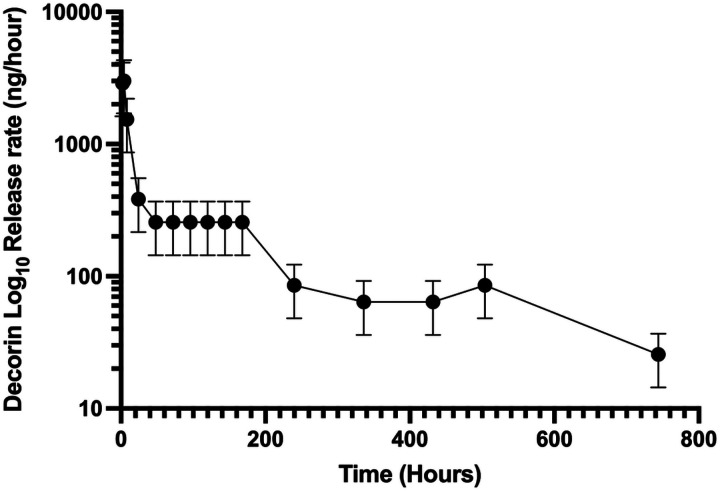
Decorin release profile from microrods. Hourly release rate (ng) of decorin from 75 mg/mL microrods over a 31 -day period shown (n = 3). It is apparent that decorin microrods exhibit burst release of decorin within first 10 hours and subsequently plateau, following first-order release behavior. The data is presented as mean ±SD.

Next, we examined the loading and in vitro release dynamics of decorin from microrods. After 4-days of passive loading, decorin microrods exhibited a loading efficiency of 98%. Release kinetics were assessed in PBS solution using aliquots of 50,000 microrods containing ~ 12.5 μg of decorin. Within the first 10 hours, decorin exhibited burst release from the microrods with more than 5.7 μg being released into solution. Following this burst release, decorin release exhibits a plateau in release from hours 24 to 168 (Fig. 2). Overall, the total hourly amount of eluted decorin decreased with time indicating concentration dependent (first-order) release kinetics from the decorin microrods. However, decorin elution was readily detectable and quantifiable over 31 days. Morphological characteristics including size and shape of the microrods remained comparable before and after loading (data not shown).

### 1.4.3. Effect of decorin microrods in ischemia-reperfusion myocardial infarction in vivo models

Decorin was loaded into microrods to achieve a more potent anti-fibrotic strategy that leveraged biophysical regulation of microstructures and the ability of decorin to sequester TGF-β1 in the post-infarct environment. Microrods were fabricated as previously described and loading according the scheme in Fig. 1.^13^ From three independent studies, decorin loading was calculated to be approximately 10.7 μg of decorin per 50,000 microrods as measured via protein quantitation (data not shown). A rodent model of ischemia-reperfusion myocardial infarction was utilized to generate a cardiac fibrosis model. All treatments– saline, microrods, decorin microrods, or free decorin were delivered into the infarct via ultrasound-guided, intramyocardial injection. For subsequent animal studies, we chose to use the higher end of observed loading (~ 12 μg per 50,000 microrods) as the injection dose for the free decorin treatment group that served as a positive control (Table 1).

**Figure 3:**
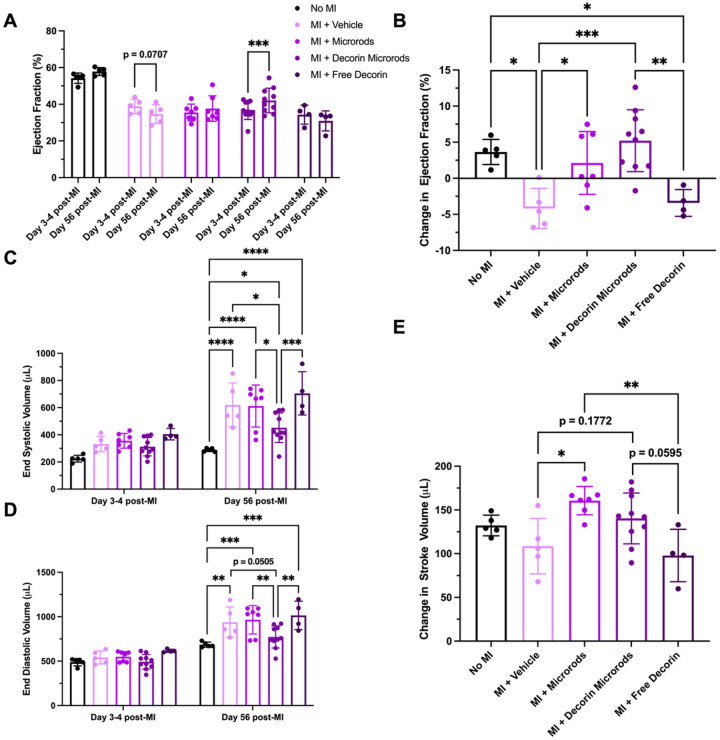
Treatment with decorin microrods improves cardiac outcomes and reduces maladaptive ventricular remodeling. Echocardiography was used to compare ejection fraction (EF) at 3–4 days after infarct and at 56 days after infarct in rats that had no MI performed (n = 5) and rats with MI that were treated with saline (n = 5), microrods (n = 7), decorin microrods (n = 10), and free decorin (n = 4). (A) The average ejection fraction (EF) at 3–4 days post-MI and EF at 56 days post-MI for each group is plotted. While saline animals show a trend for decreased EF, decorin microrod-treated animals show significant improvement in EF over the 8-week time period. (B) Rats treated with decorin microrods and microrods had a significantly higher change in EF compared to saline-treated animals. Rats treated with decorin microrods also had a significantly higher change in EF compared to those treated with free decorin. Echocardiography was used to evaluate (C) end systolic volume and (D) end diastolic volume at 3–4 days after infarct and at 56 days after infarct in all experimental groups. End systolic volume and end diastolic volume were significantly reduced in rats treated with decorin microrods compared to rats treated with saline, microrods, or free decorin. (E) Both microrod- and decorin microrod-treated animals exhibited improved change in stroke volume compared to saline and free decorin treatments. The data are presented as the mean ± SD. *p < 0.05, **p < 0.01, ***p < 0.001, ****p < 0.0001.

Microrod and decorin microrod treatment were shown to result in significant improvements in cardiac function (Fig. 3). When comparing baseline EF to EF 56 days post-MI in Fig. 3A, while saline-treated animals experienced a decline in function (*p*= 0.0707), microrod-treated animals did not experience a decline, and decorin microrod-treated animals exhibited a significant increase in EF (*p*< 0.001). Change in EF was assessed by taking the difference in EF measured at Day 3–4 post-MI and at Day 56 post-MI (Fig. 3B). Rats treated with microrods (2.12% ± 4.35%) or decorin microrods (5.21% ± 4.29%) exhibited better change in EF after 56 days than rats treated with saline (−4.18% ± 2.78%) or free decorin (−3.42% ± 1.86%). Since decorin microrods performed better than microrods and free decorin, this suggests that the ability to locally retain decorin at the infarct site through the use of the microstructures was crucial to improving the efficacy of the microrod strategy. Rats treated with decorin microrods demonstrated a significantly better change in EF at 56 days post-MI compared to both saline and free decorin (Fig. 3B, p < 0.001 and *p*< 0.01, respectively). Ventricular remodeling was also improved in rats treated with decorin microrods as evidenced by significant reduction in ESV (Fig. 3C, p < 0.05, *p*<0.05, *p*<0.001, respectively) and importantly EDV compared to rats treated with saline, microrods, and free decorin (Fig. 3D, p = 0.0505, *p*<0.01, *p*<0.01, respectively). Both microrods and decorin microrods led to a better change in stroke volume than the saline and free decorin treatments after 56 days post-MI. Microrod-treated animals had significantly higher changes in stroke volume compared to saline and free decorin groups (*p* < 0.05 and *p*< 0.01, respectively) while decorin microrods showcased a trend towards increased change in stroke volume when compared to both vehicle and free decorin (Fig. 3E, p = 0.1772, *p*= 0.0595, respectively).

**Figure 4:**
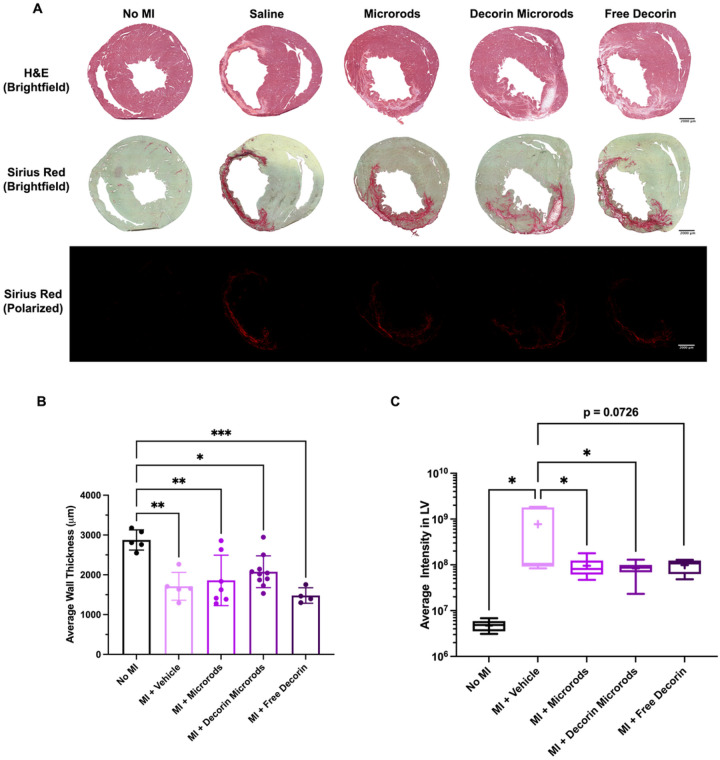
Comparison of morphology and collagen content in the various treatment groups. (A) Histological evaluation of each treatment group was performed using H&E (top) and Sirius red (brightfield: middle, cross-polarized: bottom). Scale bars = 2000 μm. (B) The average wall thickness was assessed by taking five measurements across the LV free wall in each tissue section where the LV free wall was distinguishable. Rats treated with decorin microrods showed a trend towards increased LV wall thickness compared to the free decorin group. (C) Tissue sections were stained with Sirius red and visualized under cross-polarized light. Intensity of collagen staining in the LV (including the septum) was measured. Treatment with microrods (n = 7), decorin microrods (n = 10), and free decorin (n = 4) have reduced degree of fibrosis in the left ventricle compared to saline treatment (n = 5). The data are presented as the mean ± SD. *p < 0.05, **p < 0.01, ***p < 0.001.

Histological evaluation of each treatment group was performed using H&E and Sirius red to assess LV wall thickness and degree of fibrosis (Fig. 4A). Wall thickness measurements were performed on all sections throughout the coronal plane of the heart where the LV cavity was distinguishable. Animals treated with decorin microrods (2076 ± 399 μm) exhibited trends in having greater LV wall thickness compared to those treated with saline (1711 ± 350 μm) and free decorin (1480 ±195 μm) as seen in Fig. 4B. Collagen fibrosis was assessed by quantifying the average intensity of Sirius red in the LV of 5 sections throughout each heart under cross-polarized imaging. Rats with no MI (*p*< 0.05) and rats treated with microrods (*p*< 0.05) and decorin microrods (*p*< 0.05) all had significantly reduced intensity of collagen staining in the LV compared to those treated with saline (Fig. 4C). While not statistically significant, treatment with free decorin (*p* = 0.0726) also decreased average collagen intensity in the LV compared to treatment with saline.

**Figure 5:**
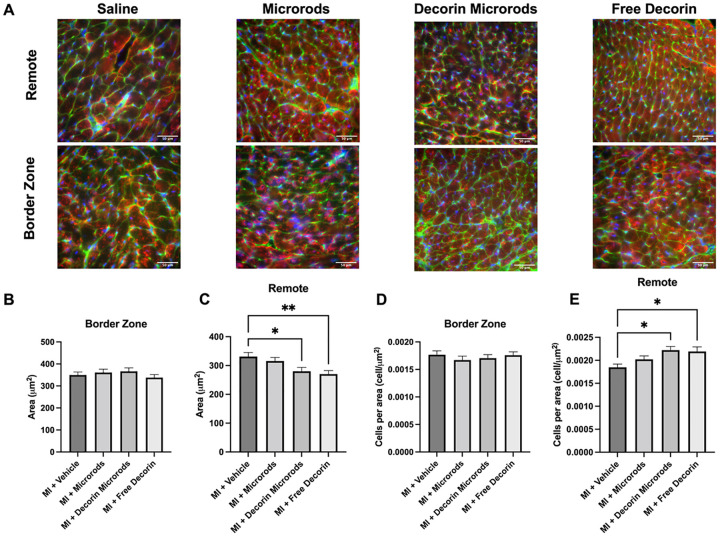
Treatment with decorin microrods and free decorin decrease cardiomyocyte hypertrophy post-MI. (A) Immunofluorescence staining for sarcomeric alpha actinin (red), WGA staining of cell membrane (green), and nuclei (blue) was performed to identify cardiomyocytes. Scale bars = 50 μm. Tissue sections were quantified for (B-C) cardiomyocyte area and (D-E) cardiomyocyte number in the border zone and remote zone. Rats treated with decorin microrods and free decorin exhibited decreased cardiomyocyte area and increased cardiomyocyte number in the remote zone compared to rats treated with saline (n = 4 for all groups). The data are presented as the mean ± SEM. *p < 0.05, **p < 0.01.

**Figure 6:**
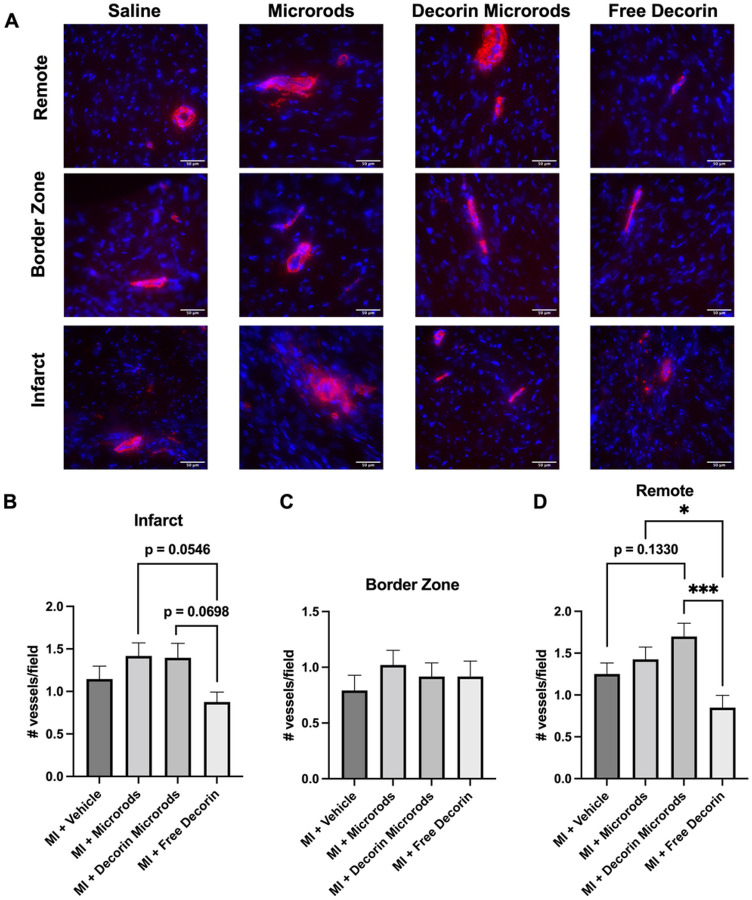
Analysis of arteriole presence post-treatment. (A) Immunofluorescence staining for alpha smooth muscle actin (red) and nuclei (blue) was performed to identify arterioles. Scale bars = 50 μm. (B-D) Tissue sections were quantified for arteriole number in the infarct, border zone, and remote zone. Trends that indicate increased number of arterioles in the microrod groups compared to free decorin are apparent (n = 4 for all groups). The data are presented as the mean ± SD. *p < 0.05, **p < 0.01, ***p < 0.001.

We also investigated the impact of decorin microrods on cardiomyocyte and endothelial cell behaviors. Given that microrods were observed to be retained in cardiac tissue for at least 8 weeks (Figure S3), we hypothesized that there could be additional long-term benefits bestowed by the presence of these structures. A crucial compensatory mechanism after myocardial injury is hypertrophic growth of cardiomyocytes.^50^ Hypertrophy was assessed by immunofluorescence staining for sarcomeric alpha actinin, cell membrane, and nuclei in the border zone and remote zone (Fig. 5A). Three sections from throughout the heart were assessed (n = 4 animals per group). While no differences in cardiomyocyte area were observed between groups in the border zone, reduced cardiomyocyte area in the remote zone was identified in decorin microrod (*p*< 0.05) and free decorin-treated animals (*p*< 0.01) compared to saline-treated animals (Fig. 5B–C). Accordingly, we observed an increase in cardiomyocytes per area in the remote zone for decorin microrod (*p*<0.05) and free decorin (*p*<0.05) groups compared to the saline group (Fig. 5D–E). Further morphometric analysis of cardiomyocytes indicated the observed smaller areas in decorin microrod and free decorin groups were due to proportional reduction in dimensions of the cells as no change in eccentricity was found (Figure S4). Studies were performed to identify if an increase in vascular density was also responsible for the observed improvements in cardiac function brought about by the decorin microrod treatment. Arteriole number was assessed by immunofluorescence staining for alpha smooth muscle actin and nuclei in the infarct, border zone, and remote zone (Fig. 6A). Three sections from throughout the heart were assessed (n = 4 animals per group). Results indicate that after 56 days post-MI, there are trends toward increased arteriole presence in both the microrod and decorin microrod groups compared to the free decorin in both the infarct (Figs. 6B–D, p = 0.0546 and *p* = 0.0698, respectively) and remote zones (Figs. 6B–D, p<0.05 and *p*< 0.001), respectively. Further, the decorin microrod group showed a trend in improved arteriole density compared to the vehicle group in the remote region (Fig. 6D, p = 0.1330). Together, this data potentially indicates some therapeutic effect of the microrods on vascular potential post-MI.

## Discussion

In this work, we investigated the capacity for microrods to locally deliver decorin to the infarct and modulate the post-infarct environment to dampen pathophysiological responses that occur post-MI. Our results demonstrate that decorin-loaded microrods release decorin for up to a month and can significantly improve cardiac function and attenuate deleterious ventricular dilatation, cardiac fibrosis, and hypertrophy in chronic models of ischemia-reperfusion MI.

An exciting area in therapeutic biomaterials research focuses on leveraging the mechanosensing machinery of cells to elicit specific phenotypes and behaviors.^7,51,52^ The ability to reliably dictate cellular responses can be a powerful tool in addressing pathological responses that result from cardiovascular diseases, such as heart failure.^7^ While injections of bulk polymers may provide the myocardium with mechanical support to improve cardiac function and delay LV dilatation, biophysical regulation via micro- and nanostructure cues can lead to reprogramming of resident cells to create microenvironments that are more amenable to tissue repair and regeneration.^51^ Our lab has previously shown the utility of high aspect ratio microstructures made from polypropylene and polyethylene glycol (PEG) to discourage myofibroblast transition.^11,12^ However, developing therapeutic strategies should aim to employ materials that have biocompatibility, exhibit biodegradation, and possess tunable properties for facile translation to the clinic. As such, next generation microrods were fabricated from HA, a naturally occurring polysaccharide that is implicated in wound healing responses.^53–55^ Advantages of employing bioactive and biodegradable materials such as HA as opposed to bioinert materials include the potential for long-acting therapeutic benefits - as HA degrades, the released oligosaccharide degradants can stimulate angiogenic processes.^55,56^ HA microrods were shown to outperform other materials in attenuating fibrotic response in vitro and in improving cardiac function in in vivo models of ischemia-reperfusion MI.^13^ These observed benefits are likely due to additional biochemical effects bestowed by the HA material itself.

Not only do polymeric microrods have the capacity lend mechanical strength to the myocardial wall and modulate fibroblast behaviors through mechanical regulation, but they can be formulated to release bioactive factors. We have successfully utilized PEG microrods to locally deliver E-domain peptide and β-NGF to injured tissue in myocardial infarction and tibial fracture models.^14,15^ To enhance efficacy of our microrod platform for applications in post-MI therapy, we investigated the ability for microrods to locally deliver decorin, a SLRP with both antifibrotic and antioxidant attributes. The core protein of decorin has two binding sites for TGF-β1 and binds collagen through its leucine rich repeat region.^57,58^ Several studies have implicated decorin in playing an important role in post-infarct remodeling and others have demonstrated how decorin treatment can mitigate adverse outcomes in various models of cardiovascular disease.^35,37, 59–61^

The results from our described in vivo experiments support these observations about the ability of decorin to improve cardiovascular outcomes after injury. While microrods improved change in ejection fraction after 56 days post-MI, decorin microrods caused the greatest improvement in cardiac function compared to all other groups, including saline and free decorin. The fact that decorin microrods performed better than microrods and free decorin individually, points to the importance of the decorin being retained in the infarct region by the microrods to achieve the optimal therapeutic effects from the combination strategy (Fig. 3). The improvements in cardiac function observed in both microrod and decorin microrod groups may likely be due to reduced LV wall stress due to the mechanical support provided by the polymer microrod injection. This is in line with what was observed in other studies where LV function improved in response to reduced LV volume and wall stress due to increased LV wall thickness resulting from biopolymer injection.^62^ Similarly, in our study decorin microrods showed a trend towards increased average LV wall thickness compared to saline and free decorin groups (Fig. 4B). Based on in vitro release data (Fig. 2) and the fact that HA microrods remain visually intact after 8 weeks within the myocardium (Figure S3), it is expected that decorin is primarily released passively from the microrods as opposed to from microrod degradation.

The presence of HA microrods after 8 weeks is not surprising as it has been shown that methacrylated HA-based hydrogels, such as our microrods, degrade enzymatically as the hydrolytic degradation sites are sterically hindered.^63^ Hyaluronidases are present at low amounts in the heart both before and after injury^64^, explaining the slow degradation of these microrods. However, our group and others have shown that when exposed to higher concentrations of hyaluronidases, these HA hydrogels will readily degrade.^13,65,66^ The presence and stability of HA microrods in the myocardium may pose benefits to long-term myocardial remodeling. Previous work has shown that similar HA hydrogels improved maintenance of wall thickness, compared to more rapidly (hydrolytically) degradable HA hydrogels with similar mechanical properties otherwise. Further, these stable enzymatic HA hydrogels elicited less inflammatory cues from the surrounding cells compared to their degradable counterparts.^63^ The immunomodulatory role that these HA microrods have within the infarct microenvironment still requires further examination, but similar HA hydrogels have been reported to elicit minimal inflammation and favorable cytocompatibility.^67^ The long-term presence of HA microrods may play a beneficial role within the myocardium remodeling, as the extended stability of similar HA gels has led to prolonged reduction in LV volume compared to degradable systems.^63^ We have also previously seen that HA microrods do not affect the contractility of cardiomyocytes.^13^ The long-term effects of HA microrods and similar HA hydrogel systems in the myocardium have not been well explored and requires further longitudinal studies to understand the myriad of cell-material and cell-cell interactions that HA systems affect.

While it was apparent that bulk cardiac metrics are enhanced in both microrod groups compared to vehicle and free decorin groups, the incorporation of decorin was hypothesized to also bestow favorable changes at the cellular level to achieve a microenvironment that is conducive to wound healing. Similar to findings from Faust *et al*. in studies utilizing decorin gene transfer, both decorin microrod and free decorin groups had decreased fibrosis (Fig. 4C) and cardiomyocyte hypertrophy compared to saline (Fig. 5).^36^ Interestingly however, only rats treated with decorin microrods exhibited improved cardiac function and ventricular remodeling outcomes (Fig. 3). Given the differences in therapeutic outcomes regarding ejection fraction and LV volumes after 56 days post-MI between the two decorin groups, it points to the presence of microrods as playing a role in the improved treatment efficacy. This additional therapeutic benefit bestowed by the microrods may be attributed to bulking of the ventricular wall as well as improved vascularization given the given the differences in vascular density between both microrod groups and the free decorin group (Fig. 6). As HA degrades, oligosaccharides are released which are known to promote angiogenic endothelial processes including proliferation, migration, and tube formation.^55,56^ However, because the injection occurred at the center of the infarct where analysis of capillary density could be subject to high variability due to confounding variables such as tissue distortion and compression, we opted instead to investigate arteriole presence as a surrogate marker of downstream vascularization.^68^ Thus, the presence and subsequent solubilization of microrods in the infarct zone may promote vessel formation. However, further investigations will need to be performed to provide more insight into this phenomenon.

While our results are promising, we recognize that there may exist some limitations to the work described here. The animal studies shown utilized male Sprague-Dawley rats to ensure that consistent models of HF were achieved. Of note, it has been documented that there may exist differences in post-MI left ventricular remodeling based that is influenced by sex. Prior studies have demonstrated favorable remodeling processes in females compared to males after myocardial injury that may be due to the ability to retain advantageous myocardial properties, such as reduced myocyte apoptosis and hypertrophy.^69–73^ These sex-related differences in remodeling responses post-MI may also explain why in the prior investigation using all female rats, the HA microrod group appeared to have a more pronounced improvement in cardiac performance compared to what was observed in the microrod groups in this study.^13^ To better account for differences in therapeutic response based on sex, future studies involving both male and female rats are necessary. Additionally, while power analysis shows that we were able to achieve significant power in the current study, future work will benefit from larger sample sizes. By identifying the relevant pathways and physiological responses that are affected by these microstructures, it will be possible to optimize this therapeutic strategy to achieve more holistic myocardial repair after injury.

## Conclusion

In this study, we have demonstrated the capacity for polymeric microstructures made of hyaluronic acid to achieve sustained delivery of the anti-fibrotic agent decorin which directly translated to therapeutic cardiac outcomes in preclinical models of I/R MI. Further, we have showcased the ability for a dual, biochemical and biophysical therapeutic strategy to synergistically improve cardiac and ventricular remodeling outcomes while attenuating collagen fibrosis and cardiomyocyte hypertrophy experienced following myocardial injury. Therefore, the use of decorin microrods represents a promising and novel translational strategy for cardiac treatment after MI. Applications of this biophysical platform in additional clinically relevant models including bone healing, cirrhosis, and implantable devices represents exciting prospects to further advance our ability to facilitate wound healing and tissue repair.

## Figures and Tables

**Table 1:** Experimental injection groups.

Treatment Group	Agent
No MI	None
MI + Vehicle	50 μL of 0.9% sodium chloride solution
MI + Microrods	50 μL of 50,000 microrods in 0.9% sodium chloride solution
MI + Decorin Microrods	50 μL of 50,000 decorin microrods (12 μg decorin) in 0.9% sodium chloride solution
MI + Free Decorin	50 μL of 12 μg decorin in 0.9% sodium chloride solution
